# Radiation-Related Alterations of Taste Function in Patients With Head and Neck Cancer: a Systematic Review

**DOI:** 10.1007/s11864-018-0580-7

**Published:** 2018-11-09

**Authors:** Tanaya S. Deshpande, Pierre Blanchard, Li Wang, Robert L. Foote, Xiaodong Zhang, Steven J. Frank

**Affiliations:** 1 Department of Radiation Oncology, The University of Texas MD Anderson Cancer Center, 1840 Old Spanish Trail, Box 1150, Houston, TX 77054 USA; 2 Department of Radiation Oncology, Gustave Roussy Cancer Campus, 114 rue Edouard Vaillant, 94800 Villejuif, France; 3 Department of Radiation Oncology, The University of Texas MD Anderson Cancer Center, 1515 Holcombe Blvd., Unit 1052, Houston, TX 77030 USA; 4 Department of Radiation Oncology, The Mayo Clinic, 200 First Street SW, Rochester, MN 55905 USA; 5 Department of Radiation Physics, UT MD Anderson Cancer Center, 1840 Old Spanish Trail, Houston, TX 77054 USA

**Keywords:** Taste alteration, Taste dysfunction, Head and neck cancer, Radiotherapy, Irradiation

## Abstract

Taste sensation is vital for a healthy body as it influences our food intake, acts as a defense mechanism and elicits pleasure. Majority of the head and neck cancer (HNC) patients undergoing radiotherapy suffer from altered taste function and often complain of inability to taste their food, reduced food intake, and weakness. However, there are not many studies conducted to assess this commonly reported side effect. Furthermore, clinical research on radiotherapy-induced taste alterations has proven to be difficult, considering a lack of reliable and validated study tools for assessing objective and subjective outcomes. Developing standardized tools for assessment of taste function and conducting prospective studies in larger population of HNC is the need of the hour. Taste sensation being critically important for sustenance, we need to focus on ways to preserve it. The physical properties of proton particle enable localization of the radiation dose precisely to the tumor and minimizing the exposure of the adjacent healthy tissues. By using Intensity-Modulated Proton Therapy in HNC patients, we anticipate preserving the taste sensation by reducing the dose of radiation to the taste buds.

## Introduction

Radiotherapy (RT) is a major treatment modality for patients with head and neck cancer (HNC). It can be delivered as a definitive treatment or as an adjuvant treatment after surgery [1]. Many patients are treated using high doses of RT over large areas including dentition, oral mucosa, salivary glands, maxilla, and mandible. Because of the direct or indirect effects of ionizing radiation, oral complications such as dental caries, xerostomia, oral mucositis, taste alteration, and candidiasis are frequent during and after treatment.

Taste, which allows us to sense sweet, salty, sour, bitter, and umami (i.e., savory) flavors, is an important sensation as it affects our ability to enjoy food and, thus, influences the nutritional intake. Loss or alteration of taste and the associated discomfort are frequent complaints during and after RT. Although primary tumors in the head and neck region rarely directly affect the sense of taste, its alteration is frequently reported by a majority of HNC patients [2, 3].

We, as humans, appreciate a wide variety of tastes because of our omnivorous evolutionary history and the genes we carry that allow us to sense sweet, salty, sour, bitter, and umami (i.e., savory) flavors. Taste sensation has three main functions: pleasure, defense, and sustenance. Taste is regulated by the brain stem mainly through cranial nerves (CN) VII (facial nerve) and IX (glossopharyngeal). The taste receptor cells on the taste buds contain nerve endings of CN VII or CN IX that detect chemical stimuli and transmit it to the brain stem which then interprets it [4].

Taste is a critical defense tool of humans [5]. Sour and salty tastes can be pleasant in small quantities, but when consumed in larger quantities become unpleasant to taste. The sour taste can signal rotten milk or meat and other spoiled foods, which can be dangerous to the body because of bacteria which grow in them. Additionally, sour taste signals acids, which can cause tissue necrosis. The bitter taste is almost universally unpleasant to humans and induces aversive reactions [6]. Many nitrogenous organic molecules which have a pharmacological effect on humans taste bitter. Also, numerous harmful compounds, including secondary plant metabolites, synthetic chemicals, inorganic ions, and rancid fats, do taste bitter. So, bitter taste may be considered as a warning sign against the ingestion of potential poisons. On the other hand, sweetness helps to identify energy-rich foods. Sweet taste signals the presence of carbohydrates. Since carbohydrates have a very high calorie count, they are desirable to the human body. Similarly, salt has an important role in maintaining homeostasis in our body. When the Na levels in our body fall below a certain level, our body starts craving for salt to replenish the Na levels. Because of this, salt elicits a pleasant taste in most humans.

Even though taste dysfunction functionally alters a person’s life and RT can cause alterations of taste sensation, there are not many studies conducted to assess the effect of RT on this vital function. Few studies in small groups of individuals at different time points and using different methods have been conducted previously. However, the results need to be compiled to better understand the problem and evaluate it overall.

The aim of this systematic review is to assess (1) the degree in which radiation therapy to the head and neck region alters the taste function; (2) if all the five tastes are altered to the same extent; (3) if this side effect of RT is temporary or permanent and present the natural history and extent of taste recovery; and (4) if there is an established dose-volume dysgeusia relationship in the oral cavity or tongue.

## Methods

**Fig. 1:**
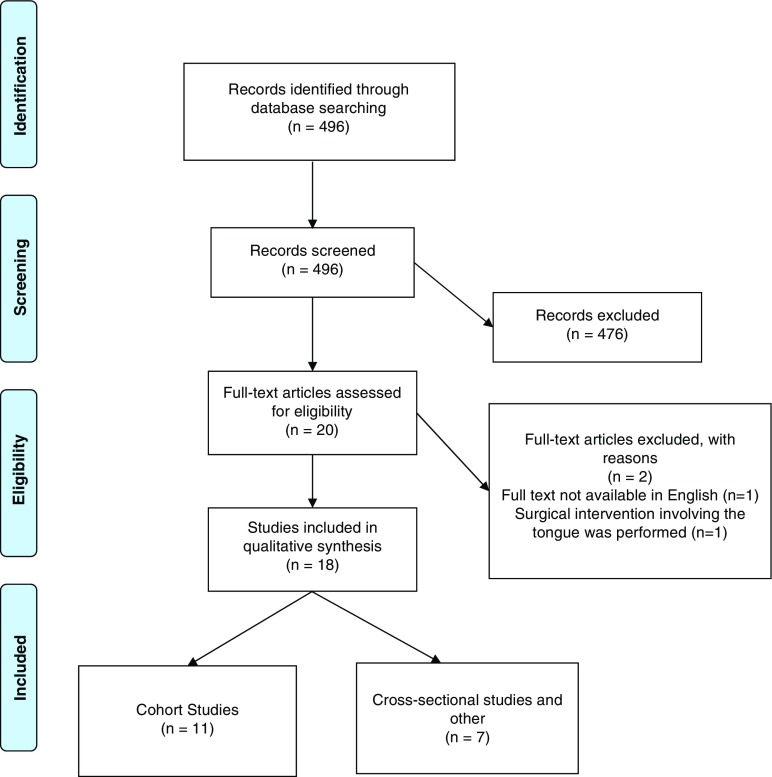
PRISMA 2009 flow diagram for “Radiation-Related Alterations of Taste Function in Patients With Head and Neck Cancer: a Systematic Review.” Based on [7].

The systematic review was performed in accordance with the Preferred Reporting Items for Systematic Reviews and Meta-Analyses (PRISMA) Statement 2009 [7]. The flow of information through the different phases of the systematic review was constructed on its basis (Fig. 1). No systematic reviews have been conducted on this subject using the PRISMA guidelines. The use of this checklist improves the reporting quality of systematic reviews and provides substantial transparency in the article selection process.

We systematically reviewed publications focusing on taste alterations among HNC patients.

### Eligibility criteria

Studies meeting specific criteria were included:

1. Published in English;
2. Prospective and retrospective cohort studies and cross-sectional studies;
3. Studies among HNC patients treated with RT with or without chemotherapy and surgery;
4. Objective taste assessment test (OTA) and/or subjective taste assessment (STA) performed before, during and after RT.

In our review, we did not restrict the time of publication.

#### Exclusion criteria

Articles were excluded if they (1) were not published in English, or (2) were done on patients other than head and neck cancer or (3) had partial or total glossectomy as an intervention.

##### Information sources and search strategies

A systematic search of the PubMed database was done in June 2018. No other sources were evaluated. The key words searched were “head,” “neck,” “cancer,” and “taste.” The search was limited to the English literature published. Titles and abstracts of the studies identified were screened for potential inclusion and selected using the pre-determined criteria. Full texts of potentially eligible studies were retrieved. The few relevant articles which full text access through PubMed did not have were accessed on ScienceDirect or through MD Anderson library.

##### Selection of studies and data extraction process

The articles retrieved were individually searched to identify the eligible studies.

Cohort studies with taste assessments performed on HNC patients prior to RT and at one or more time points during and after completion of RT were selected. Cross-sectional studies that had taste assessments performed on HNC patients previously treated with RT were also included.

Following the study selection process, we extracted data from the included studies and reported in Excel (Tables 1 and 2).

**Table 1:** Cohort studies on taste alteration among head and neck cancer patients

Reference	*N*	Study design	Location/type of primary tumor	Type of RT	Taste assessment	Time points for data collection	Results
Fernando et al. [8]	*N* = 26	PC	OC, OP, HP, L, ethmoids, ear, and Max. antrum	Not specified	Taste solutions; STA	OTL and STL at baseline and EOT; STL at 1-mo post-RT	OTL (*P* = 0.0016) and STL (*P* = 0.0001) was associated with the proportion of tongue, contained within the field of RT. No relationship between recovery of taste loss and volume of tongue irradiated.
Shi et al. [9]	*N* = 30	PC	OC, OP, NP, HP, L, and nasal vestibule	2D-RT with CT	Whole mouth technique; STA	Pre-RT; irradiation doses at 15, 30, 45, and 60 Gy	Sweet, sour, salty, and bitter tastes showed no statistical difference between the threshold at pre-RT and that at 15, 30, 45, and 60 Gy. Significantly impaired threshold of umami taste was revealed at 30 Gy (*P* < 0.05) and remained at 45 and 60 Gy (both *P* < 0.01).
Yamashita et al. [10]	*N* = 51	PC	OP, NP, HP, and others	2D-RT w/wo CT	FPD	Baseline and weekly from 1 wk to 10–12 wks after start of RT	All tastes declined on the fifth wk after the start of RT and improved on the 11th wk. The effect w/wo CT did not differ.
Sandow et al. [11]	N1 = 13; N0 = 5	PC	OP	2D-RT w/wo CT	Whole mouth technique	Before RT and at 1-, 6-, and 12-mo post-RT	There were significant elevations in thresholds for sweet, salty, bitter, and sour during radiation therapy at the 1-mo threshold test that were restored to baseline levels at 6 mo and 1 year after RT.
Yamashita et al. [12•]	*N* = 118	PC	OC, OP, NP, HP, L, CN, maxilla, NC, lymphomas, and others	Not specified	FPD	Baseline; weekly from 1st wk up to 10–16 wks; monthly from 4 mo to 14–24 mo after start of RT	In pts w/ anterior tongue irradiated, significant impairment of the threshold of all four basic tastes at 3 wks after starting RT that persisted until 8th wk (*p* < 0.05). The impairment was not significant in those w/ the tip of tongue not in the radiation field.
Mirza et al. [13]	N1 = 8; N0 = 17	PC	OP, NP, L, and SG	2D-RT	Pipette droplet; video microscopy	Baseline and 2 wks, 2-mo, and 6-mo post-RT	Sour taste was significantly affected in pts at 2-mo post-RT. At 6-mo post-RT, pts had lower taste test scores for bitter, salty, sour tastes w/ bitter, and sour, most impaired. Taste pores decreased in the RT group.
Kamprad et al. [14]	N1 = 44; N0 = 30	PC	Head and neck tumor	Not specified	Taste solutions for 4 basic tastes	Before RT, during RT, and 8 wks, 1-, 2-, 3-, and 6-mo post-RT	Gustatory disturbance was more severe in pts w/ entire tongue irradiated. The gustatory disturbances regressed within 8wks after RT in pts with partial-tongue irradiation and almost completely after 6mo in pts w/ entire-tongue irradiation
Yamashita et al. [15]	*N* = 52	PC	OP, NP, HP, and other	2D-RT w/wo CT	Whole mouth technique	Baseline; weekly from 1-wk to 10–12-wk post-RT	Umami taste declined in the 3rd wk after the start of RT and improved of the 8th wk.
Baharvand et al. [2]	*N* = 22	PC	OC, OP, NP, HP, SG, maxilla, and mandible	2D-RT w/wo CT	Whole mouth technique; STA	At baseline and 3-week post-RT	All pts had dysgeusia after RT (*p* < 0.001) and 72.2% had total taste loss. Impairment was observed mainly in salt and bitter. Subjective dysgeusia was correlated w/ objective dysgeusia w.r.t taste sensitivity.
Pavlidis et al. [16•]	*N* = 20	PC	OP, HP, L, and SG	2D-RT w/wo CT or CT only	EGM and contact endoscopy	Baseline, 3 wks after beginning of t/t and post-RT.	Elevated EGM thresholds at 3 wks seen in all pts. Complete ageusia (*P* < 0.001) was observed at the EOT. RT-treated pts had higher EGM thresholds and greater alterations in morphology and vascularization of FP than the RCT and CT pts.
Riva et al. [17•]	N1 = 30; N0 = 30	RC	NP	2D-RT/3D-RT/IMRT w/wo CT	Taste strips test (filter paper strips)	4- to 13-year post-RT	A higher percentage of hypogeusia was found in irradiated pts than the controls. Sweet, bitter, salty, and taste total score was significantly lower for IMRT, compared w/ 2D-RT/3D-CRT group.
Sapir et al. [18]	*N* = 73	PC	OP	IMRT w/ CT	QOL including STA	At baseline, 1-, 3-, 6-, and 12-mo post-RT	Severe dysgeusia was reported by 50%, 40%, 22%, and 23% of pts at 1, 3, 6, and 12 mo after t/t respectively. Significant associations were found between patient-reported severe dysgeusia and radiation dose to OC (*P* = .005) and tongue (*P* = .019)

*CT*, chemotherapy; *CS*, cross-sectional; *CN*, cervical nodes; *c/o*, complaining of; *EGM*, electrogustometry; *FPD*, filter paper disc method; *FP*, fungiform papillae; *HL*, Hodgkin’s lymphoma; *HP*, hypopharynx; *L*, larynx; *mo*, months; *LSM*; laser-scanning microscopy; *N1*, cases/patients; *N0*, controls; *NC*, nasal cavity; *NP*, nasopharynx; *NA*, not applicable; *OC*, oral cavity; *OP*, oropharynx; *P*, pharynx; *PC*, prospective cohort; *pts*, patient; *RC*, retrospective cohort; *RT*, radiotherapy; *SG*, salivary glands; *STA*, subjective taste assessment; *t/t*, treatment; *wk*, week; *w/w0*, with or without

**Table 2:** Cross-sectional studies on taste alteration among head and neck cancer patients

Reference	*N*	Study design	Location/type of primary tumor	Type of RT	Taste assessment	Time points for data collection	Results
Mossman et al. [3]	*N* = 13	CS	OC, OP, NP, HP, L, and SG	2D-RT	Forced-choice, three stimulus drop technique; STA	1- to 7-year post-RT	69% of the pts had measurable taste loss; salt and bitter affected most, and sweet and sour affected least. Taste intensity responsiveness most severely affected for salt and bitter.
Schwartz et al. [19]	*N*1 = 15; *N*0 = 23	CS	OC, OP, NP, SG, CN, and neck	2D-RT	Whole mouth technique; STA	1- to 19-year post-RT	Near normal suprathreshold taste intensity perception in pts who have received head and neck RT. Type and taste intensity disturbance may be related to the age of the pt.
Maes et al. [20]	*N* = 73	CS	OC, OP, NP, HP, and SG	2D-RT	Whole mouth technique; STA	Baseline, 2, 6, and 12–24 mo after RT	Loss of taste was most pronounced after 2 mo after RT. Bitter and salt qualities were most impaired. Gradual recovery seen during the first year after RT. Partial taste loss persisted 1–2 years. after RT.
Just et al. [21]	*N*1 = 12, *N*0 = 12	CS	OP, HP, L, and SG	RCT	LSM; filter paper strips and EGM.	During or after RCT	Pts c/o taste disorders during RCT exhibited a significant decrease of taste function assessed with both natural and electric stimuli. Thicker epithelia and smaller areas of the taste pores in pts compared with healthy subjects. In 30% pts, no taste pores detectable.
McLaughlin et al. 2013	*N* = 92	CS	OC, P, L, paranasal cavity, and others	Surgery and/or RT, RT w/ CT w/wo surgery	Whole mouth technique	3 months to more than 28 years after completion of t/t	85 of 92 participants had some measurable taste dysfunction. Confusion between bitter and sour and the inability to discriminate among the different concentrations of the sweet solutions seen. Statistically significant weight loss was associated with dysgeusia.
Mossman et al. [22]	*N* = 27	NA	OC, OP, NP, HP, L, HL, and SG	2D-RT	Forced-choice, three stimulus drop technique; STA	Baseline, during RT, 1 mo post-RT and 1 year post-RT	The bitter and salt qualities showed earliest and greatest impairment and the sweet quality the least. Scaling impairment was most severe for bitter and salt tastes. Scaling impairment occurred before changes in either detection or recognition thresholds.

*CT*, chemotherapy; *CS*, cross-sectional; *CN*, cervical nodes; *c/o*, complaining of; *EGM*, electrogustometry; *FPD*, filter paper disc method; *FP*, fungiform papillae; *HL*, Hodgkin’s lymphoma; *HP*, hypopharynx; *L*, larynx; *mo*, months; *LSM*, laser-scanning microscopy; *N1*, cases/patients; *N0*, controls; *NC*, nasal cavity; *NP*, nasopharynx; *NA*, not applicable; *OC*, oral cavity; *OP*, oropharynx; *P*, pharynx, *PC*, prospective cohort; *pts*, patient; *RC*, retrospective cohort; *RT*, radiotherapy; *SG*, salivary glands; *STA*, subjective taste assessment; *t/t*, treatment; *wk*, week; *w/w0*, with or without

##### Data items

The following information was extracted from the included studies: number of participants; location of primary tumor; type of taste assessment test; type of RT delivered; time points at which the taste test was performed; and the results of the studies, as reported.

##### Risk of bias in individual studies

This systematic review did not include a meta-analysis on the outcomes. Thus, the assessment of the risk of bias was performed at the study level and the body of evidence was not presented.

The study quality of the prospective studies was assessed using the Newcastle-Ottawa Scale (NOS, Table 3), which scores the quality of non-randomized studies [23]. For a cohort study, NOS has three domains: (1) the selection of the cohort; (2) the comparability of the cohorts; and (3) the quality of outcome for cohort study. NOS identifies quality with “stars.” A maximum of one star for each item within the selection and outcome categories and a maximum of two stars for comparability can be given in the NOS. The total maximum score is 9 stars. Ratings of ≥  7 were high; 4 to 6, moderate; and 4 or less, low quality, were used in the present study. A modified version of the NOS scale (Table 4) was used to assess the quality of the cross-sectional studies. The total maximum score was 6 stars. Ratings of ≥  5 were high; 3 to 4, moderate; and 3 or less, low quality. The average NOS scores for the cohort and cross-sectional studies were calculated separately.

**Table 3:**
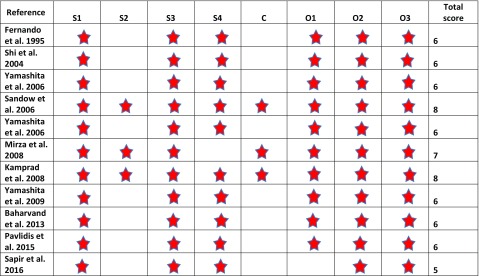
Newcastle-Ottawa Scale (NOS) for cohort studies

*S1*, representativeness of the exposed cohort (exposed to HN radiation) to the general population; *S2*, selection of the non-exposed cohort (HN region not exposed to radiation) from the same population; *S3*, ascertainment of radiation exposure; *S4*, taste alteration not present at start of study; *C*, comparability of cohorts; *O1*, assessment of outcome; *O2*, length of follow-up adequate for taste alteration to occur; *O3*, adequacy of follow-up

**Table 4:**
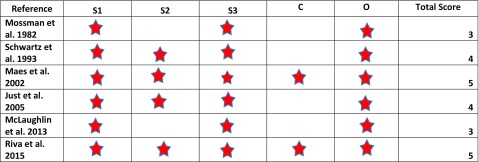
Modified Newcastle-Ottawa Scale (NOS) for cross-sectional studies

*S1*, representativeness of the sample to the general population; *S2*, selection of the non-exposed sample (HN region not exposed to radiation) from the same population; *S3*, ascertainment of radiation exposure; *C*, comparability of exposed and unexposed; *O1*, assessment of taste alteration

### Synthesis of results

A quantitative synthesis would not have been appropriate because of the variation in the type of taste assessment test across individual studies. A systematic narrative synthesis was provided with the information presented in the text and tables to summarize and explain the methodology and findings of the included studies. The narrative synthesis explored the relationships and the findings both within and among the included studies.

## Results

### Study selection

Our search strategy identified 496 articles to be considered for inclusion. The titles and abstracts that did not fulfill our inclusion criteria were excluded at this stage itself. After screening titles and abstracts, 20 articles were selected to be evaluated in full text for eligibility. Of the 20 articles retrieved, 2 were excluded as one of them did not have full text available in English while the other included patients that underwent surgical resection of tongue. Eighteen studies fulfilled all our inclusion and exclusion criteria and were included in the systematic review.

### Study characteristics

Out of 18 studies included, 12 were cohort studies and five were cross-sectional studies. One study did not have a specific study design with data collected prospectively in one study group and cross-sectionally in the other. Characteristics, methods, and results for the eighteen included studies are summarized in Table 1 (cohort studies) and Table 2 (cross-sectional studies). A wide variety of taste assessment tests were used across the studies to asses taste alteration among HNC patients. Few studies did not specify the test but mentioned the concentrations of the taste solutions used [8, 14]. Some of the studies also conducted STA of taste function parallel with the objective tests performed [2, 3, 8, 9, 19, 20, 22]. One study used a structured questionnaire alone to analyze patient-reported outcomes (PROs) related to taste function [18].

### Taste assessment test methods

The basis of all the taste assessment tests is similar which includes determination of taste thresholds by application of different taste solutions on the patients’ tongue and asking the patient to identify the type of taste. The tests that were seen to be commonly used in the studies for OTA were (1) whole mouth technique; (2) forced-choice three stimulus drop technique; (3) filter paper disc (Taste strips) method; and (4) electrogustometry (EGM). Different concentrations of the taste agents were prepared for these tests. The patient was exposed to the solutions in ascending order of their concentrations till the patient guessed the taste correctly. The concentration at which the patient was able to identify the taste rightly was recorded. This was repeated for all four or five taste types. In the whole mouth technique, the patient was asked to swish the test solution in his mouth for few seconds, spit it out, and guess its taste. The filter paper disc method used filter paper strips to apply the taste agent to the patient’s tongue. For the forced-choice three stimulus drop technique, the patient was exposed to three consecutive drops of the test solution (2 drops of distilled water and 1 drop of test solution), and was asked to identify the drop that tasted differently from the other two drops and what was the taste of that drop. For EGM, electric stimuli instead of chemical stimulus were used for taste function assessment.

Based on the average NOS scores calculated, the overall quality of the cohort studies and the cross-sectional studies was moderate (Average NOS score for cohort studies = 6.36 and average NOS score for cross-sectional studies = 4).

### Risk of bias within studies

The cross-sectional studies included assessed different groups of patients at various time points following RT. Most of these studies did not account for individual differences in treatment protocols and taste experience [3, 20, 22]. These studies were with highly heterogeneous sample, which made it difficult to draw conclusions on the relationship between time and taste dysfunction [16•, 19, 20]. For example, in the study by Maes et al. [20], a higher proportion of patients in group 1 underwent surgery. Also, two of the cross-sectional studies do not specify whether the enrolled cases treated with surgery underwent glossectomy procedure or not [19, 20]. Therefore, a prospective study with a more homogenous sample and precisely reported treatment interventions could help determine how taste is altered over time.

In the study by Fernando et al. [8], only STA performed at 1-month post-RT and no OTA was used to report results. Although no significant relationship between the volume of tongue or parotid tissue and taste recovery was found, it should be noted that only 21 of 26 patients were evaluated. However, recovery in two patients, both of whom were treated by a wedge pair technique to spare the contralateral area of tongue, was detected. This recovery occurred despite the exit dose to the contralateral tongue from the radiation beams [8].

The results of McLaughin [24•] study are limited by a number of procedural and design flaws. The sample size was small and highly heterogeneous relative to the number of variables examined. As a result, conservative estimations were required for several variables. The participants were drawn from a convenience sample. Controlling for potential mediator variables such as tobacco use, medications, or history of medical conditions associated with taste changes (e.g., Alzheimer disease, head injury) would have enhanced the generalizability of the study results. In addition, weight change was calculated based on the first weight recorded in the clinic chart. Head and neck cancer survivors often present with weight loss. Change in body mass index may have more accurately represented the relationship between weight change and taste impairment [24•].

In the study by Just et al. [21], the exact time point (time elapsed since RT or if the patient was receiving RT at that point when the tests were performed) is not specified. Also, taste loss observed may be due to chemotherapy because anterior tongue was not in the RT volume. Taste pores were covered by epithelial cells which may explain the taste loss [21].

### Results of individual studies

On reviewing the literature, it was found that majority of the patients (70–100%) experienced partial or total taste loss during RT. Impairment was observed in all five tastes. Irrespective of RT to the tip of the tongue, bitter and salty tastes were found to be most severely affected while sweet taste was least affect [2, 3, 20, 22]. All tastes declined at 4th to 5th week after the start of RT and improved on the 11th week [10]. Regardless of the taste quality, maximum impairment in taste was seen in patients during the fourth to sixth week. Maes et al. [20] reported the loss of all taste types to be most pronounced at 2 months following RT. Recovery from taste loss was seen to start as early as 4–5 weeks after completion of radiotherapy. Around 6–12 months after RT completion, recovery from dysgeusia was reported in most patients [11, 20]. However, partial taste loss of all taste types persists for 1–2 years after treatment as reported in one of the studies [14, 20]. Maes et al. [20] also performed a subgroup analysis of taste changes based on age of the patients. However, no significant difference in the prevalence of taste loss between the different age groups was found. In contrast to this, Schwartz et al. [11] in their study in 1993 suggests that the type and taste intensity disturbance likely to be manifested in a cancer patient irradiated to the head and neck may be related to the age of the patient. This study also provides evidence for near normal suprathreshold taste intensity perception in patients who have received head and neck irradiation.

Taste intensity responsiveness of the patients was affected before changes in the detection and recognition thresholds. As for thresholds, scaling impairment was most severe for bitter and salt taste qualities [22].

The clinical impairment pattern of umami taste is different from that of the other four basic tastes in HNC patients during radiotherapy. Shi et al., in their study, found a significantly impaired threshold of umami taste at 30 Gy (*P* < 0.05) that remained throughout the following treatment (at 45 and 60 Gy, both *P* < 0.01). This was not observed for the remaining four tastes [9]. In contrast, a maximum reduction of gustatory ability for four basic tastes was seen after a local dose of 40–60 Gy to the tongue area [14].

The severity of dysgeusia is related to the radiation dose to the oral cavity and the tongue. A significant association was also found between patient-reported severe dysgeusia and radiation dose to the oral cavity (*P* = .005) and tongue (*P* = .019) [18]. Some studies may report a discrepancy between frequency of subjective awareness of taste loss and measurable taste loss [3]. This discrepancy may be due to adaptation of the patient to the sensory loss. Thus, objective assessment may be more reliable as compared to the subjective assessment of taste function.

A significant association was also seen between the extent of dysgeusia and the proportion tongue in the radiation field. Entire-tongue irradiation group showed a far more distinct gustatory disturbance as compared to the patients with partial-tongue irradiation. But there was no evidence to suggest any relationship between recovery of taste loss and volume of tongue irradiated [14]. In another study by Yamashita, it was seen that unless the anterior part of the tongue was irradiated, even though the base of the tongue was included in the high-dose region, acute taste loss was not observed during and after RT. When the anterior part of the tongue was irradiated, even low radiation doses such as 20 Gy resulted in temporal taste loss. Acute taste loss pattern was independent of radiation dose [12•].

The taste alteration during RT is believed to be caused due to atrophy of taste buds rather than nerve injury. Just et al. [21] compared the microscopic structure of the tongue of the patients treated with chemoradiotherapy (CRT) and suffering from taste disorders to that of the healthy subjects. In the patients, he found thicker epithelia and smaller areas of the taste pores compared with healthy subjects. In 30% of those patients, no taste pores were detectable. This was supported by the study on rats by Yamashita et al. [10]. The number of taste bud cells among the irradiated rats diminished almost completely by 6 days after irradiation and returned to about 80% on the 19th day. [10]. Sandow et al. [11] also stated that time-course for recovery of taste in his study suggested that the site of radiation damage is at the level of the taste cells, rather than the nerves. If there was nerve damage, it is unlikely that taste would be restored at 6 months post-RT.

Most of the CRT patients with significant decrease of taste function as determined by chemical stimuli also experienced subjective symptoms [3, 9, 19]. The subjective dysgeusia was correlated with objective dysgeusia with respect to taste sensitivity; nevertheless, such correlation was absent with respect to the concentration level of gustatory qualities, meaning the higher the level of subjective dysgeusia, the lower the taste sensitivity [2].

Radiotherapy alone causes greater disorders of taste compared with CT and combined CRT [16•]. RT patients exhibited higher electrogustometry thresholds and greater alterations in the morphology and vascularization of the fungiform papillae than the RCT and CT patients [16•]. In the study by McLaughin [24•], taste dysfunction was a persistent problem across all categories of treatments, sites, and stages among HNC patients. Participants who reported the loss of one or more specific taste modality performed poorly on the taste test. However, participants could not accurately predict which taste was most severely impaired.

## Discussion

Radiation therapy to the head and neck region significantly affects taste function. The taste intensity responsiveness and the taste recognition and/or detection thresholds are all impaired. All the five taste types are seen to decline around the fifth week after the start of RT. Bitter and salty tastes are affected the most while the sweet taste is the least affected.

Recovery of taste function may occur as early as 4 to 5 weeks after the completion of RT. Complete recovery of taste function following RT is still not quantified or reported. Whether the damage caused to the taste buds is temporary or permanent is still unclear. Partial taste loss is seen to be prevalent even 20 years after completion of RT.

The observed impairment in taste function is due to radiation-induced atrophy of the taste buds. The extent of dysgeusia is found to be proportional to the radiation dose to the tongue. Sparing the anterior one third of the tongue and/or as much of the anterior oral cavity as possible from RT could help reduce the loss or altered sense of taste, reduce malnutrition and gastrotomy tube dependency during and after treatment, and may improve patient’s overall quality of life and survival.

The study conducted by Rosenthal et al. reported that there is an increased beam path dose to alternate non-target structures that may result in clinical toxicities that were uncommon with previous 3D-RCT [25]. However, from the articles reviewed, we were unable to conclude a significant difference in the taste function of the patients treated with various RT techniques.

Although Intensity-Modulated Photon Radiotherapy (IMRT) has a wonderful record for tumor control and survival for patients with HNC while reducing xerostomia, it is associated with increased rates of several different toxicities related to dose deposited in the beam path [26–28]. A relatively simple yet highly effective method to reduce radiation-induced toxicity is through the physical displacement of adjacent tissues away from the tumor using a customized oral stent. The use of oral stents has proven to delay emergence of mucositis or cause no mucositis but its role in preservation of taste function has not yet been explored [29, 30].

The use of proton RT for cancer management has increased in recent times. The charge and mass of protons allow lower exit doses than photons enabling the localization of the radiation dose precisely to the tumor. This allows the exposure of the adjacent healthy tissue to be reduced minimizing the side effects [31•]. Research to date shows Intensity-Modulated Proton Therapy (IMPT) to be a safe method for treatment associated with low rates of acute toxicities. In a comparative study of PROs of IMPT versus IMRT for Oropharyngeal Cancer, reduced symptom burden in the IMPT patients was seen during the subacute recovery phase following treatment. Among the top 11 symptoms, changes in taste and appetite during the subacute and chronic phases favored IMPT (all *P* < 0.048) [31•].

By using proton RT for treating head and neck cancer patients, we anticipate protecting the taste buds and salivary glands preserving taste sensation. Furthermore, we hypothesize that reduced radiation dose to normal tissue from proton RT will result in less damage to the taste buds and adjacent salivary glands compared to that caused using x-rays or photon therapy. Also, the flow rate and composition of saliva will be altered to a lesser extent compared to that during photon RT.

## Limitations

The whole mouth method cannot detect subtle difference in taste threshold compared with forced-choice stimulus drop method or the disc method. Nine out of the 18 studies in the review have a very small sample size (*n* ≤ 30). Thus, the results of these studies can only be considered preliminary. Future research is required in this field to establish significant associations and conclusions. Also, only six studies had control groups with healthy patients unexposed to radiation. Thus, comparison of taste assessment results to accurately assess the effect of RT on taste can only be reported with the use of a control group.

Furthermore, there is no standard test to assess taste alteration. Even within a specific type of test, say whole mouth technique, there were multiple variations seen among different studies. Each investigator has used different taste solutions as well as different concentrations of the taste solutions. Thus, meta-analysis of findings was not possible.

## Conclusion

Taste is a critical function of survivability. Taste impairment has profound effects on patients’ quality of life because of its crucial role in body’s defense mechanism, inciting pleasure and necessity for sustainability. It is associated with weight loss due to reduced appetite and may even lead to decrease survival due to affected nutritional status. Altered taste perception in HNC patients is usually overlooked by clinicians as this aspect does not represent any acute life-threatening events. With proton therapy, we expect patients to have improved taste perception and retain their healthy appetite, thus enhancing their nutritional status and preventing weight loss. Hence, using proton therapy for head and neck cancer treatment can be highly beneficial to HNC patients through a positive impact on their quality of life and long-term outcomes.
